# Diagnostic Value of Endotracheal Tube-Aspirate Soluble Triggering Receptor Expressed on Myeloid Cells-1 Concentration for Neonatal Ventilator-Associated Pneumonia

**DOI:** 10.3389/fped.2021.664801

**Published:** 2021-08-25

**Authors:** Jian Zhou, Jingqian Zhou, Yan Hong, Youcheng Wang, Hailong Lin, Leting Huang

**Affiliations:** ^1^Department of Pediatrics, The First People's Hospital of Yongkang, Jinhua, China; ^2^Department of Pediatrics, The Central Hospital of Wuhan, Wuhan, China; ^3^Department of Pediatrics, Jinhua People's Hospital, Jinhua, China; ^4^Department of Pediatrics, The Second Affiliated Hospital and Yuying Children's Hospital of Wenzhou Medical University, Wenzhou, China

**Keywords:** triggering receptor expressed on myeloid cells-1, ventilator, pneumonia, ventilator-associated pneumonia, newborn

## Abstract

**Background:** Soluble triggering receptor expressed on myeloid cells-1 (sTREM-1) is regarded as a biological marker of infection. We aimed to evaluate the diagnostic value of endotracheal tube (ETT)-sTREM-1 concentration in neonatal ventilator-associated pneumonia (NVAP), to explore the difference of (ETT)-sTREM-1 between preterm and full-term, and to investigate the influence of extrapulmonary infection on (ETT)-sTREM-1 concentration.

**Methods:** In this multicenter, controlled clinical trial of 60 preterm and 33 full-term neonates on mechanical ventilators, we measured concentrations of ETT-aspirate and serum sTREM-1, serum C-reactive protein, and serum procalcitonin, as well as white blood cell count. We initially divided cases into eight groups, based on three categories: preterm of full-term; NVAP or non-NVAP; and extrapulmonary infection present or absent. Groups were compared, and logistic regression analysis and receiver operating characteristic (ROC) analysis was performed to determine diagnostic value.

**Results:** The mean gestational age (± standard deviation) of preterm and full-term neonates was 28.9 ± 2.2 weeks and 39.5 ± 1.7 weeks, respectively, and 32/60 were male. The ETT-aspirate sTREM-1 concentration was higher in NVAP cases than in non-NVAP cases, irrespective of extrapulmonary infection. ROC analysis revealed that ETT-aspirate sTREM-1 concentration had an area under the curve (AUC) of 0.986 and a cutoff value of 228.0 pg/ml (sensitivity, 94.3%; specificity, 96%) in preterm neonates; the same values in full-term neonates were 0.938 and 245.5 pg/ml (sensitivity, 100%; specificity, 93.7%), respectively. The optimal combination of indicators was ETT-aspirate sTREM-1 and serum C-reactive protein concentration. All indicators were present at lower levels on days 8 and 10 of ventilation in neonates who ultimately recovered than in those who did not.

**Conclusions:** ETT-aspirate sTREM-1 and serum C-reactive protein concentrations may be useful for the diagnosis of NVAP.

## Background

Neonatal ventilator-associated pneumonia (NVAP) is a type of lung infection that occurs in newborns who are on mechanical ventilators. As such, it typically affects critically ill newborns in neonatal intensive care units. NVAP is a major source of increased length of hospitalization, illness, and death, with a mortality rate of 20–30% (1–3). The diagnosis of NVAP is difficult and not yet standardized. A common method for diagnostic confirmation of ventilator-associated pneumonia (VAP) is sampling of lung sputum or tracheal aspirates for culture, although its clinical applicability is limited, especially in infants (4). Furthermore, the Centers for Disease Control (CDC) criteria refer to infants younger than 1 year, with no criteria specific for newborns (5).

Soluble triggering receptor expressed on myeloid cells-1 (sTREM-1) is a transmembrane glycoprotein expressed on the inflammatory cells including neutrophils, macrophages and monocytes. It is up-regulated in effector cells infected by bacteria and fungi. In critically ill adults with VAP, the elevated levels of sTREM-1 were found in endotracheal tube-aspirate (ETT-aspirate) and exhaled ventilator condensate (EVC) (6–9). Biological markers of infection, such as soluble triggering receptor expressed on myeloid cells-1 (sTREM-1), may improve the accuracy of the diagnosis of VAP (6, 10). However, we are not aware of reports on its use in newborns. Therefore, in this study, our aim was to determine the diagnostic value of endotracheal tube (ETT)-aspirate sTREM-1 concentration in NVAP.

## Methods

### Trial Design and Ethics Statement

This was an open, controlled clinical trial, approved by The First People's Hospital of Yongkang's institutional ethics committee (approval number: ykyy2018-03). After obtaining ethics approval, we registered the trial in the Chinese Clinical Trial Registry (approval number: ChiCTR1900020564), and recruited the first participants in January 2019.

### Participants

**Figure 1:**
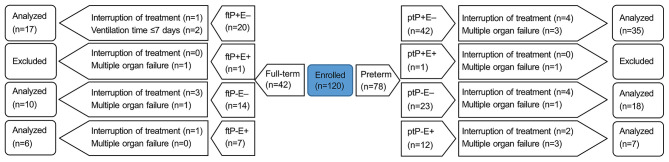
A flow diagram of the trial. Neonates were divided into eight groups based on three categories: preterm (pt) or full-term (ft); NVAP (P+) or non-NVAP (P–); and extrapulmonary infection present (E+) or absent (E–). NVAP, neonatal ventilator-associated pneumonia.

Case inclusion criteria were as follows: (1) newborns, including preterm and full-term; and (2) mechanical ventilation initiated within 48 h of birth. Case exclusion criteria were as follows: (1) >28 days after birth; (2) infectious lung disease before mechanical ventilation, including neonatal pneumonia, meconium aspiration syndrome, or either local or systemic infection affecting the lungs; (3) mechanical ventilation initiated after 48 h; (4) multiple organ failure. Predetermined withdrawal criteria were as follows: (1) not meeting the inclusion criteria at any time during the trial; (2) total time of mechanical ventilation ≤ 7 days; or (3) any other reason, medical or otherwise, such as adverse drug reactions or impaired participant's interests, for which we deemed it in the subject's best interest to be withdrawn from the trial. We enrolled 120 subjects in three hospitals: 51 in The First People's Hospital of Yongkang, 37 in Jinhua People's Hospital, and 32 in The Central Hospital of Wuhan. Objective to explore the difference of (ETT)-sTREM-1 between preterm and full-term, and to investigate the influence of extrapulmonary infection on (ETT)-sTREM-1 concentration, we divided these subjects into eight groups, based on three categories: preterm (pt) or full-term (ft); NVAP (P+) or non-NVAP (P–); and extrapulmonary infection present (E+) or absent (E–). Thus, the eight groups were: ptP+E–, ptP+E+, ptP–E–, ptP–E+, ftP+E–, ftP+E+, ftP–E–, and ftP–E+. See Figure 1 for a flow diagram of subjects enrolled and completing the trial for each of the groups.

We assessed NVAP according to criteria in the CDC's 2016 device-associated module on pneumonia events (11): (1) temperature >38°C or <36°C; (2) white blood cell count >12,000/mm^3^ or <4,000/mm^3^; (3) purulent/increased/change in character of respiratory secretions; (4) positive tracheal/endotracheal tube cultures; (5) signs of respiratory distress, such as shortness of breath, rapid breathing, or abnormal breathing sounds upon auscultation; (6) increased oxygen requirement/ventilator demand; (7) at least two serial chest X-rays revealing sustained/worsening shadowing (infiltrates/consolidations); and (8) positive cultures obtained directly from lung tracheal aspirates. In this study, we diagnosed a patient with NVAP based on criterion (7) and at least three of criteria (1)–(6); we also diagnosed a patient with NVAP based on criterion (8).

Ventilation modes applied with pressure-controlled synchronized intermittent mandatory ventilation with pressure support (SIMV-PC/PS). Initial ventilatory settings were: VT = 4 mL/kg; PEEP, (5.47 ± 0.51) cmH_2_O; and peak inspiratory pressure (PIP), (18.06 ± 1.18) cmH_2_O; and FiO_2_ to maintain a saturation of 90–95%. If necessary, dopamine 2–10 μg/kg min for haemodynamic support, midazolam 1–3 μg/kg min for analgesia, and fentanyl 1–2 μg/kg h for sedation. Piperacillin/tazobactam injection was used as antibiotics empirically, then it was adjusted to the sensitive antibiotics with the results of bacterial culture.

### Interventions

We measured six indicators every 2 days from the 4th to the 10th day on the ventilator: (1) white blood cell count; (2) serum C-reactive protein concentration; (3) serum procalcitonin concentration; (4) serum sTREM-1 concentration; (5) ETT-aspirate sTREM-1 concentration; and (6) bacterial culture of the serum and/or ETT-aspirate.

Three other indicators were recorded as a routine measure: (1) daily vital signs, including body temperature, respiration, pulse, and oxygen saturation; (2) daily ventilator parameters, including fraction of inspired oxygen, positive end-expiratory pressure, and/or peak inspiratory pressure; (3) chest X-rays taken on the first day of the trial, when pneumonia was confirmed, and reviewed every 3 days. All the patients who suffered an infection received antibiotic treatment.

ETT-aspirate samples were collected according to a standard procedure (12). Briefly, the neonate was placed supine with the head turned to the right, to predominantly sample the right lung. We instilled 1 ml/kg of 0.9% NaCl into the endotracheal tube. After two ventilator cycles, the suction catheter was gently inserted 0.5 cm beyond the tip of the ventilator tube, and the airway fluid was aspirated into a sterile specimen trap (ETT-aspirate Trap; Vygon SA, Écouen, France) with 50 mmHg of negative pressure. ETT-aspirate samples were excluded from further analysis if there was visible blood staining or if there covered volume was <30% of the instilled volume. This procedure was repeated with the head turned to the left, to predominantly sample the left lung. An aliquot of sterile isotonic saline solution was added to reach 2 ml of total quantity. The minimum the amount of absorption back is 0.8 ml, it needs 1.2 ml saline solution to be added to reach 2 ml. Therefore, the ratio of the amount of absorption back to saline solution is 1:1.5. On this basis, all recovered samples were added with sterile isotonic saline solution at the ratio of 1:1.5. Then 2 ml of the mixed volume was used for further detection. After collection, ETT-aspirate specimens were homogenized and centrifuged at 1,000 × g for 5 min. Cell-free supernatants were removed and the pH was measured using a potentiometric micromethod analyzer (ABL 725 Plus; Radiometer Medical ApS, Copenhagen, Denmark). The remaining supernatant volume was immediately frozen at −70°C for later testing.

sTREM-1 was detected by sandwich enzyme-linked immunosorbent assay (Human TREM-1 Quantikine ELISA Kit; R&D Systems, Inc., Minneapolis, MN, USA).

### Statistical Analysis

Data processing and statistical analysis was performed using SPSS Statistics for Windows, Version 17.0 (SPSS Inc., Chicago, IL, USA). Groups were compared by means of two-tailed *t*-tests; repeated measures ANOVA and ROC (receiver operating characteristic) analysis were performed specifically for groups ptP+E– and ftP+E–. We constructed multivariate logistic regression models incorporating different pairs of markers, and univariate logistic regression models were used when assessing one marker at a time. Continuous data were displayed as the mean ± standard deviation.

## Results

### Patient Demographics and Pathogens

We analyzed 60 preterm and 33 full-term neonates, none of which tested positive for both pneumonia and extrapulmonary infections; thus analysis was conducted only on six groups,due to multiple organ failure, none of ptP+E+ or ftP+E+ was included. See Figure 1 for numbers enrolled in, and excluded from, each group. The majority of preterm neonates (32/60) analyzed were male. The mean gestational age of preterm and full-term neonates was 28.9 ± 2.2 and 39.5 ± 1.7 weeks, respectively.

The diseases identified in preterm neonates included neonatal respiratory distress syndrome, necrotizing enterocolitis, neonatal hyperbilirubinemia, neonatal cerebral ischemia, and neonatal septicemia. The diseases identified in full-term neonates included neonatal hypoxic ischemic encephalopathy, neonatal hyperbilirubinemia, congenital lactic acidosis, neonatal septicemia, and congenital megacolon.

### Indicators

**Figure 2:**
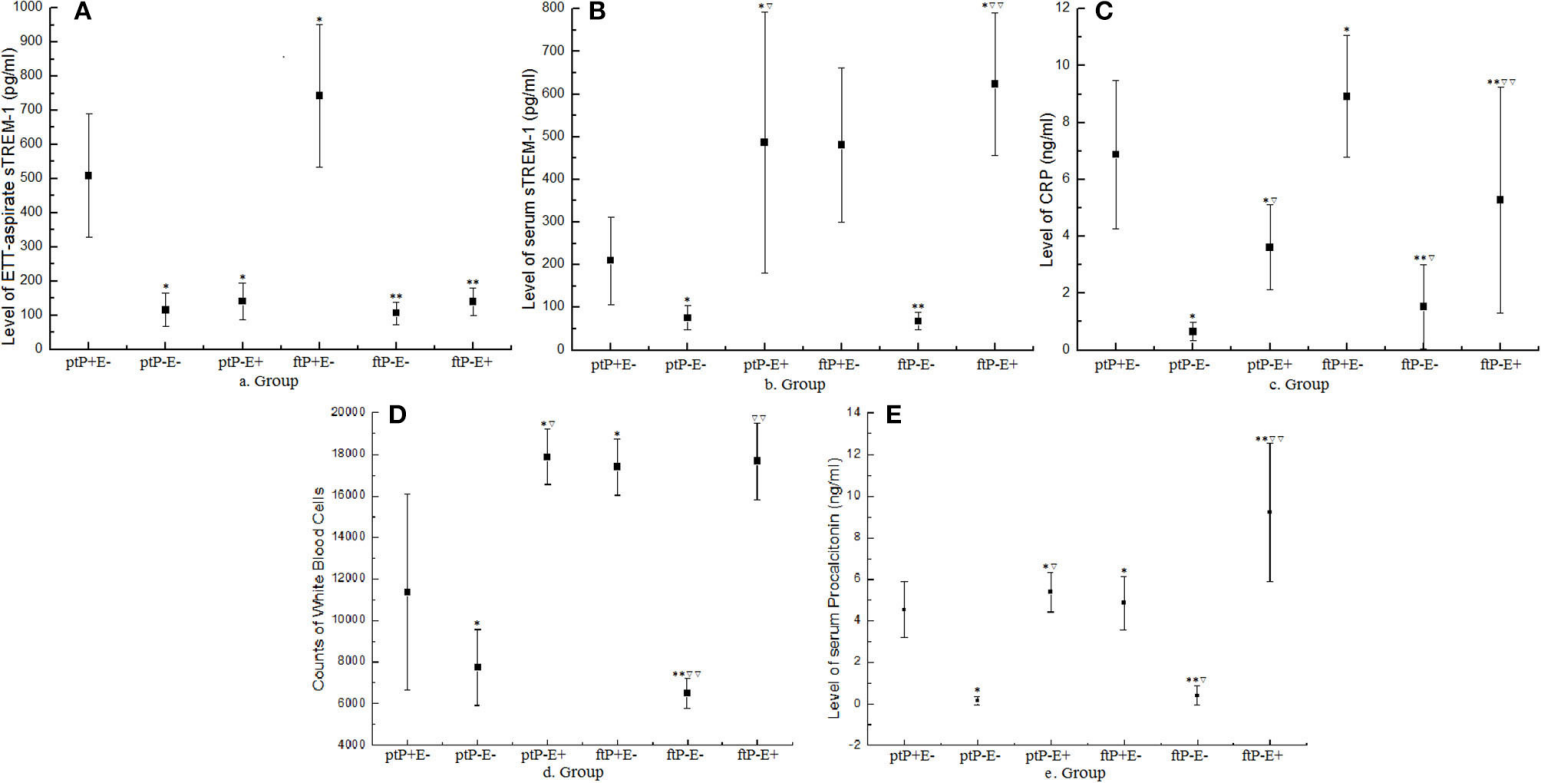
Values of indicators on the fourth day on the mechanical ventilator. Dots represent mean values and error bars represent the standard deviation. Groups are based on three categories: preterm (pt) or full-term (ft); NVAP (P+) or non-NVAP (P–); and extrapulmonary infection present (E+) or absent (E–). ETT, endotracheal tube; sTREM-1, soluble triggering receptor expressed on myeloid cells-1; CRP, C-reactive protein; NVAP, neonatal ventilator-associated pneumonia. *P < 0.05 when compared with the ptP+E– group; ^▿^*P* < 0.05 when compared with the ptP–E– group; **P < 0.05 when compared with the ftP+E– Group; ^▿▿^*P* < 0.05 when compared with the ftP–E– Group.

In terms of day 4th on the ventilator of ETT-aspirate sTREM-1 levels (Figure 2A), there was a difference between the ptP+E– (508.25 ± 180.70 pg/ml) and ptP–E– (115.50 ± 49.01 pg/ml) groups (*t* = 9.0135, *P* < 0.05), as well as between the ptP+E– and ptP–E+ (140.89 ± 53.19 pg/ml) groups (*t* = 5.2855, *P* < 0.05), but not between the ptP–E– and ptP–E+ groups (*t* = 1.1370, *P* > 0.05). There was a difference between the ftP+E– (742.57 ± 207.76 pg/ml) and ftP–E– (106.00 ± 33.01 pg/ml) groups (*t* = 12.3704, *P* < 0.05), as well as between the ftP+E– and ftP–E+ (138.64 ± 40.86 pg/ml) groups (*t* = 6.9711, *P* < 0.01), but not between the ftP–E– and ftP–E+ groups (*t* = 1.7552, *P* > 0.05). There was also a difference between the ptP+E– and ftP+E– groups (*t* = 4.1855, *P* < 0.05), but between neither the ptP–E– and ftP–E– groups (*t* = 0.5458, *P* > 0.05), nor the ptP–E+ and ftP–E+ groups (*t* = 0.0843, *P* > 0.05).

Regarding day 4th serum sTREM-1 levels (Figure 2B), there were differences between the ptP+E– (209.85 ± 102.64 pg/ml) and ptP–E– (75.50 ± 27.93 pg/ml) groups (*t* = 5.4276, *P* < 0.05), between the ptP+E– and ptP–E+ (487.24 ± 305.65 pg/ml) groups (*t* = 4.4207, *P* < 0.05), as well as between the ptP–E– and ptP–E+ groups (*t* = 5.8549, *P* < 0.05). There were differences between the ftP+E– (480.58 ± 180.83 pg/ml) and ftP–E– (67.75 ± 20.47 pg/ml) groups (*t* = 5.8906, *P* < 0.05), as well as between the ftP–E– and ftP–E+ (623.69 ± 167.12 pg/ml) groups (*t* = 10.6367, *P* < 0.05), but not between the ftP+E– and ftP–E+ groups (*t* = 1.6963, *P* > 0.05). There was also a difference between the ptP+E– and ftP+E– groups (*t* = 6.8963, *P* < 0.05), but between neither the ptP–E– and ftP–E– groups (*t* = 0.7749, *P* > 0.05), nor the ptP–E+ and ftP–E+ groups (*t* = 0.9721, *P* > 0.05).

Upon comparing day 4th ETT-aspirate and serum sTREM-1 levels, there were differences within each group (group ptP+E–: *t* = 8.4948, *P* < 0.05; group ptP–E–: *t* = 3.0084, *P* < 0.05; group ptP–E+: *t* = 2.9537, *P* < 0.05; group ftP+E–: *t* = 3.9289, *P* < 0.05; group ftP–E–: *t* = 3.1141, *P* < 0.05; and group ftP–E+: *t* = 6.9060, *P* < 0.05).

With respect to day 4th white blood cell count (Figure 2C), there were differences between the ptP+E– (11,358.46 ± 4,704.71/mm^3^) and ptP–E– (7,728.83 ± 1,818.86 /mm^3^) groups (*t* = 4.7774, *P* < 0.05), between the ptP+E– and ptP–E+ (17,867.51 ± 1,340.04/mm^3^) groups (*t* = 6.9304, *P* < 0.05), as well as between the ptP–E– and ptP–E+ groups (*t* = 9.8140, *P* < 0.05). There were differences between the ftP+E– (17,374.20 ± 1,361.47/mm^3^) and ftP–E– (6,493.33 ± 733.33/mm^3^) groups (*t* = 29.4420, *P* < 0.05), as well as between the ftP–E– and ftP–E+ (17,658.22 ± 1,849.32/mm^3^) groups (*t* = 23.3148, *P* < 0.05), but not between groups ftP+E– and ftP–E+ (*t* = 0.6450, *P* > 0.05). There were also differences between groups ptP+E– and ftP+E– (*t* = 5.3034, *P* < 0.05), as well as between groups ptP–E– and ftP–E– (*t* = 2.5348, *P* < 0.05), but not between groups ptP–E+ and ftP–E+ (*t* = 0.2363, *P* > 0.05).

With reference to day 4th serum C-reactive protein levels (Figure 2D), there were differences between the ptP+E– and ptP–E– groups (*t* = 10.0254, *P* < 0.05), groups ptP+E– and ptP–E+ (*t* = 3.1819, *P* < 0.05), groups ptP–E– and ptP–E+ (*t* = 6.1642, *P* < 0.05), groups ftP+E– and ftP–E– (*t* = 7.1073, *P* < 0.05), groups ftP+E– and ftP–E+ (*t* = 2.8448, *P* < 0.05), groups ftP–E– and ftP–E+ (*t* = 2.7430, *P* < 0.05), groups ptP+E– and ftP+E– (*t* = 2.8028, *P* < 0.05), and groups ptP–E– and ftP–E– (*t* = 2.4435, *P* < 0.05).

Concerning day 4th serum procalcitonin levels (Figure 2E), there was a difference between the ptP+E– and ptP–E– groups (*t* = 19.4827, *P* < 0.05), groups ptP–E– and ptP–E+ (*t* = 15.1137, *P* < 0.05), groups ftP+E– and ftP–E– (*t* = 9.5044, *P* < 0.05), groups ftP+E– and ftP–E+ (*t* = 7.7982, *P* < 0.05), groups ftP–E– and ftP–E+, groups ptP+E– and ftP+E– (*t* = 1.9879, *P* < 0.05), groups ptP–E– and ftP–E– (*t* = 0.8090, *P* < 0.05).

Bacterial culture results from serum and ETT-aspirate indicated the presence of various bacteria, as summarized in Table 1.

**Table 1:** Bacterial culture results of serum and ETT-aspirate in each group.

Group^*^	**Bacteria from blood**	**Bacteria from ETT-aspirate**
ptP+E–	*S. epidermidis* (1)^†^	*K. pneumoniae* (6), *P. aeruginosa* (4), *A. baumannii* (3) *E. coli* (3), and *C. albicans* (1)
ptP–E–	None	None
ptP–E+	*A. baumannii* (1) *E. coli* (3)	None
ftP+E–	*C. albicans* (1)	*K. pneumoniae* (4), *P. aeruginosa* (2), *A. baumannii* (1), and *E. coli* (1)
ftP–E–	*S. epidermidis* (1)	None
ftP–E+	*A. baumannii* (1), *E. coli* (2), and *P. aeruginosa* (1)	None

* *Groups are based on three categories: preterm (pt) or full-term (ft); NVAP (P+) or non-NVAP (P–); and extrapulmonary infection present (E+) or absent (E–)*.

† *Numbers in parentheses indicate the number of neonates testing positive for the bacterial species. ETT, endotracheal tube; S. epidermidis, Staphylococcus epidermidis; A. baumannii, Acinetobacter baumannii; E. coli, Escherichia coli; C. albicans, Candida albicans; P. aeruginosa, Pseudomonas aeruginosa; K. pneumoniae, Klebsiella pneumoniae; NVAP, neonatal ventilator-associated pneumonia*.

### Receiver Operating Characteristic Analysis of Indicators for Pneumonia

**Figure 3:**
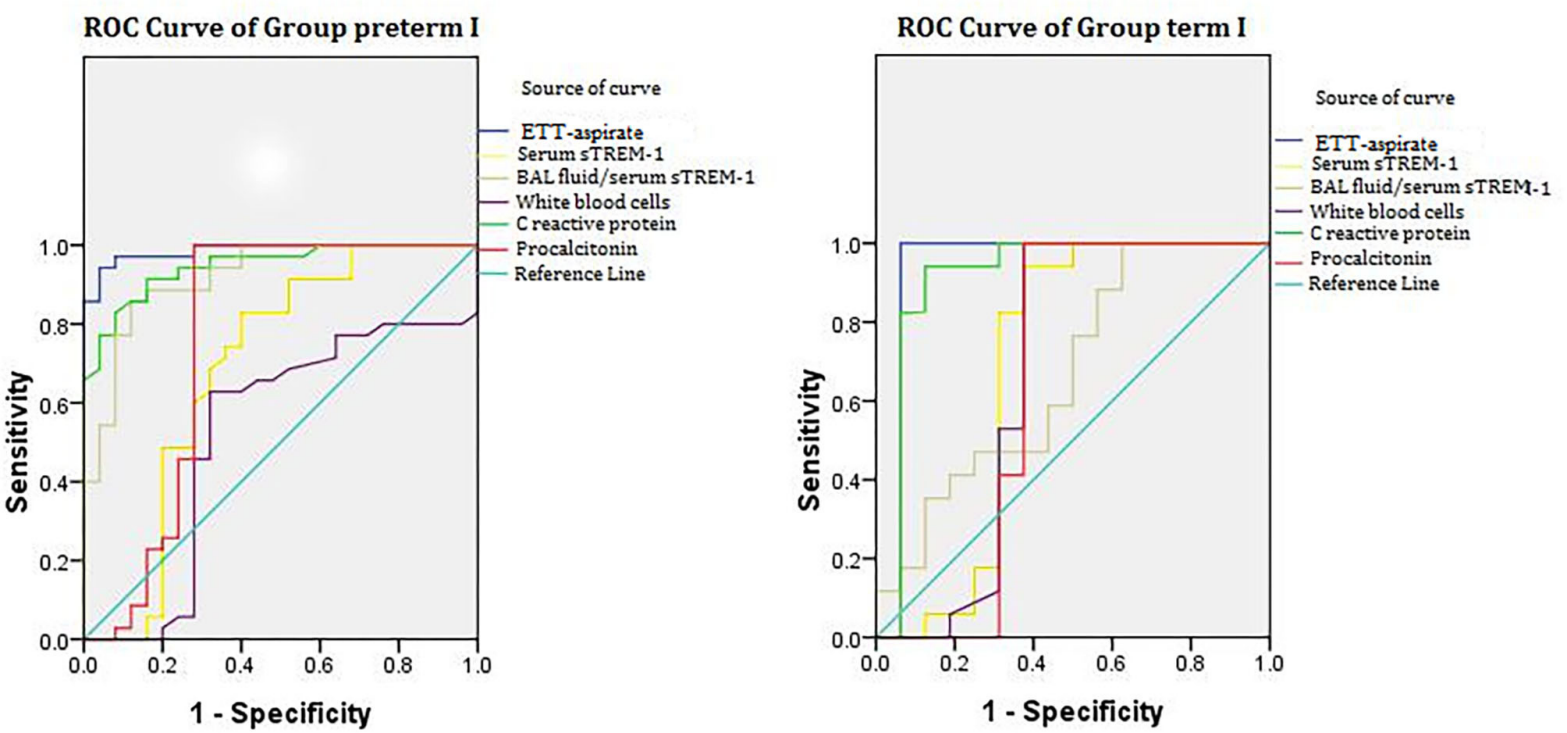
ROC curve of groups ptP+E– and ftP+E–. ROC analysis performed in groups ptP+E– (17 cases) and ftP+E– (35 cases). Groups are based on three categories: preterm (pt) or full-term (ft); NVAP (P+); and extrapulmonary infection absent (E–). ROC, receiver operating characteristic; ETT, endotracheal tube; sTREM-1, soluble triggering receptor expressed on myeloid cells-1; NVAP, neonatal ventilator-associated pneumonia.

Figure 3 illustrates the ROC analysis performed to determine the diagnostic value of indicators in groups ptP+E– (17 cases) and ftP+E– (35cases). Table 2 summarizes the results in terms of area under the curve (AUC), cutoff value, sensitivity, and specificity. For both groups, the AUCs of ETT-aspirate sTREM-1, serum sTREM-1, and serum C-reactive protein were statistically significant (*P* < 0.05). The AUCs of serum procalcitonin and ETT-aspirate/serum sTREM-1 were only statistically significant for group ptP+E– (*P* < 0.05). The AUC of white blood cell count was not statistically significant for either group (*P* > 0.05).

**Table 2:** ROC analysis of indicators in groups ptP+E– and ftP+E–.

**Indicator**	**AUC**	**95% CI**	**Cutoff value**	**Sensitivity**	**Specificity**	**Indicator**	**AUC**	**95% CI**	**Cutoff value**	**Sensitivity**	**Specificity**
ETT-aspirate sTREM-1	0.986^*^	0.920–1.000	228.0 pg/ml	94.3%	96.0%	ETT-aspirate sTREM-1	0.938^*^	0.937–1.000	245.5 pg/ml	100.0%	93.7%
Serum sTREM-1	0.689^*^	0.535–0.843	105.5 pg/ml	82.9%	60.0%	Serum sTREM-1	0.688^*^	0.474–0.901	129.0 pg/ml	94.1%	62.5%
ETT-aspirate sTREM-1	0.720	0.650–0.890	1.4926	94.3%	68.0%	ETT-aspirate/serum sTREM-1	0.669	0.481–0.857	1.2591	100.0%	37.5%
White blood cells	0.521	0.361–0.681	10,250.00/mm^3^	62.9%	68.0%	White blood cells	0.669	0.450–0.888	11,700.00/mm^3^	100.0%	62.5%
Serum C-reactive protein	0.947^*^	0.896–0.998	2.88 ng/ml	91.4%	84.0%	Serum C-reactive protein	0.915^*^	0.903–1.000	6.25 ng/ml	94.1%	87.5%
Serum procalcitonin	0.762^*^	0.608–0.917	1.24 ng/ml	100.0%	72.0%	Serum procalcitonin	0.651	0.424–0.877	2.28 ng/ml	100.0%	62.5%
Group ptP+E–^†^	Group ftP+E–^†^										

* *P < 0.05*.

† *Groups are based on three categories: preterm (pt) or full-term (ft); neonatal ventilator-associated pneumonia (P+); and extrapulmonary infection absent (E–). ROC, receiver operating characteristic; AUC, area under the curve; CI, confidence interval; ETT, endotracheal tube; sTREM, soluble triggering receptor expressed on myeloid cells-1*.

We constructed multivariate logistic regression models incorporating different pairs of markers. Using prediction probability as diagnostic indicator, we determined diagnostic cutoff values from the ROC curve. The AUCs of the two-marker models were all larger than that of any single marker model. The optimal combination was ETT-aspirate sTREM-1 and serum C-reactive protein concentration, for which AUC (95% confidence interval [CI]), sensitivity, and specificity was 0.968 (0.909–0.999), 98.9, and 72.7%, respectively, in group ptP+E–, and 0.926 (0.918–1.000), 95.6 and 92.4%, respectively, in group ftP+E–.

### Repeated Measures ANOVA of Indicators for Ventilator-Associated Pneumonia

In the ptP+E– and ftP+E– groups, 19/35 and 11/17 neonates, respectively, recovered. All indicators were more favorable at the third and fourth time points in neonates who recovered than in those who did not, in each group (all *P* < 0.05; Table 3). We observed no differences at any time point for serum or ETT-aspirate sTREM-1 concentrations between the ptP+E– and ftP+E– groups (all *P* > 0.05), and none of the values changed over time in ptP+E– or ftP+E– neonates who did not recover (all *P* > 0.05).

**Table 3:** Repeated measures ANOVA of indicators in groups ptP+E– and ftP+E–.

	**White blood cells (/mm3)**				**C-reactive protein (ng/ml)**				**Procalcitonin (ng/ml)**				**Serum sTREM-1 (pg/ml)**				**ETT-aspirate/ sTREM-1 (pg/ml)**			
**Group**	**Time point 1**	**Time point 2**	**Time point 3**	**Time point 4**	**Time point 1**	**Time point 2**	**Time point 3**	**Time point 4**	**Time point 1**	**Time point 2**	**Time point 3**	**Time point 4**	**Time point 1**	**Time point 2**	**Time point 3**	**Time point 4**	**Time point 1**	**Time point 2**	**Time point 3**	**Time point 4**
Not recovered	11,580 ± 4,500	11,930 ± 5,010	12,590 ± 5,300	12,330 ± 5,250	7.08 ± 2.02	7.28 ± 2.08	7.53 ± 2.08	7.90 ± 2.31	4.38 ± 1.66	4.63 ± 1.46	4.88 ± 1.34	4.92 ± 1.39	247.50 ± 90.79	254.69 ± 88.84	256.44 ± 94.68	250.31 ± 84.86	539.38 ± 197.43	556.75 ± 182.77	565.44 ± 182.92	573.31 ± 177.87
Recovered	11,530 ± 5,000	11,010 ± 4,670	8,630 ± 2,450^*^	7,010 ± 1,150^*^	8.44 ± 2.11	6.97 ± 2.18	2.57 ± 1.33^*^	0.84 ± 0.34^*^	4.73 ± 1.06	3.50 ± 1.41^*^	1.36 ± 0.76^*^	0.52 ± 0.50^*^	266.68 ± 85.15	241.68 ± 84.91	116.37 ± 45.52^*^	72.42 ± 22.62^*^	550.89 ± 165.96	507.21 ± 170.46	233.84 ± 94.29^*^	134.79 ± 63.34^*^
F time (*P*-value)		7.981 (0.004)				100.978 (<0.001)				88.338 (<0.001)				63.752 (<0.001)				87.239 (<0.001)		
F group (*P*-value)		3.497 (0.070)				22.669 (<0.001)				31.587 (<0.001)				10.344 (0.003)				15.838 (<0.001)		
F time × group (*P*-value)		15.984 (<0.001)				147.894 (<0.000)				147.511 (<0.001)				66.862 (<0.001)				113.199 (<0.001)		
Group ptP+E–																				
	**White blood cells (/mm3)**				**C-reactive protein (ng/ml)**				**Procalcitonin (ng/ml)**				**Serum sTREM-1 (pg/ml)**				**ETT-aspirate/ sTREM-1 (pg/ml)**			
**Group**	**Time point 1**	**Time point 2**	**Time point 3**	**Time point 4**	**Time point 1**	**Time point 2**	**Time point 3**	**Time point 4**	**Time point 1**	**Time point 2**	**Time point 3**	**Time point 4**	**Time point 1**	**Time point 2**	**Time point 3**	**Time point 4**	**Time point 1**	**Time point 2**	**Time point 3**	**Time point 4**
Not recovered	17,450 ± 1,290	17,430 ± 990	18,000 ± 1,160	18,020 ± 1,370	7.98 ± 1.95	8.13 ± 2.38	8.57 ± 2.03	8.63 ± 1.93	4.67 ± 1.01	4.88 ± 0.81	4.92 ± 0.85	5.00 ± 0.76	544.00 ± 74.78	593.67 ± 56.24	592.33 ± 74.31	595.50 ± 85.77	871.67 ± 136.67	883.83 ± 158.84	855.83 ± 117.65	864.00 ± 114.10
Recovered	18,900 ± 1,150^*^	17,000 ± 2,190	11,950 ± 1,650^*^	7,880 ± 850^*^	9.70 ± 2.07	7.78 ± 1.42	2.62 ± 0.71^*^	0.96 ± 0.33^*^	5.40 ± 1.39	3.95 ± 1.42	1.46 ± 0.85^*^	0.40 ± 0.33^*^	588.27 ± 155.74	464.00 ± 105.66^*^	211.09 ± 68.39^*^	104.45 ± 26.93^*^	866.64 ± 185.71	712.73 ± 124.08^*^	331.18 ± 89.63^*^	168.64 ± 52.96^*^
F time (*P*-value)		104.120 (<0.001)				46.757 (<0.001)				35.608 (<0.001)				31.000 (<0.001)				86.218 (<0.001)		
F group (*P*-value)		38.075 (<0.001)				21.652 (<0.001)				24.802 (<0.001)				47.733 (<0.001)				38.547 (<0.001)		
F time × group (*P*-value)		133.049 (<0.001)				63.639 (<0.001)				44.595 (<0.001)				43.299 (<0.001)				77.903 (<0.001)		
Group ftP+E–																				

* *Compared to not recovered, P < 0.05. Groups are based on three categories: preterm (pt) or full-term (ft); neonatal ventilator-associated pneumonia (P+); and extrapulmonary infection absent (E–). Indicator values are the mean ± standard deviation. ANOVA, analysis of variance; sTREM-1, soluble triggering receptor expressed on myeloid cells-1; ETT, endotracheal tube*.

## Discussion

sTREM-1, a member of the immunoglobulin superfamily, is a soluble TREM-1 that is upregulated when neutrophils are exposed to bacteria, but not during noninfectious inflammatory diseases, which suggests that sTREM-1 may be a specific marker for infectious diseases (13–15). Previous research has demonstrated that sTREM-1 yielded a high sensitivity (>95%) and specificity (>85%) in systemic inflammatory response syndrome patients (16, 17), and may be useful as C-reactive protein and procalcitonin of diagnostic value for sepsis severity and helpful for prognostic assessment (18), or more useful than them with regard to sepsis diagnosis in adult and pediatric patients (19, 20). sTREM-1 can be measured directly in human body fluids, including serum, pleural effusion, sputum, and urine, during infections (17, 21). Urine sTREM-1 may play a role in the early diagnosis of sepsis and sepsis-related acute kidney injuries (22), but not a sufficient biological marker for infection of the urinary tract (21). A study found Serum sTREM-1, PCT, and CRP levels each have a role in the early diagnosis of sepsis. Serum sTREM-1, with the highest sensitivity and specificity of all indicators studied, is especially notable (23). And then another study found in patients with ventilator-associated pneumonia, serum levels of sTREM-1 plus the pulmonary infection score are useful for diagnosis, and procalcitonin levels plus the pulmonary infection score are useful for prognostic assessment (24). The concentration of sTREM-1 has been demonstrated to rise in the ETT-aspirate or BAL fluid of VAP patients (6–9), including a pediatric research of VAP patients after cardiac surgery, but none about neonatal VAP (25). One study found Plasma levels of sTREM-1 did not change significantly in either VAP and non-VAP patient group. While in controls concentrations of sTREM-1 in bronchial lavage fluid did not change significantly over time, in patients who developed VAP levels of sTREM-1 in bronchial lavage fluid increased toward the diagnosis of VAP (26). Different from the above results, in several studyies ETT-aspirate or bronchoalveolar lavage fluid sTREM-1 failed to serve as an effective biomarker of culture positive VAP in levels did not effectively categorize patients as VAP positive or VAP negative (27, 28). The discrepancy between the results need more and further researches to explain.

In this study, we investigated the usefulness of sTREM-1 concentration in serum and ETT-aspirate for the diagnosis of NVAP. We assessed preterm and full-term neonates separately, due to their different physiological characteristics. Among both of these groups, the ETT-aspirate sTREM-1 concentration was higher, on average, in those diagnosed with NVAP without extrapulmonary infection than in those not diagnosed with NVAP, irrespective of the presence of extrapulmonary infection. Among those diagnosed with NVAP without extrapulmonary infection, ETT-aspirate sTREM-1 concentrations were higher, on average, in full-term than in preterm neonates, which may indicate a stronger ability to express sTREM-1 by the immune system of the former.

Among both preterm and full-term neonates, the mean serum sTREM-1 concentration was higher in both those not diagnosed with NVAP but indeed with extrapulmonary infection, and in those diagnosed with NVAP and no extrapulmonary infection, than in those not diagnosed with NVAP or extrapulmonary infection. However, only among full-term neonates was the mean serum sTREM-1 concentration higher in those not diagnosed with NVAP but indeed with extrapulmonary infection, than in those diagnosed with NVAP and no extrapulmonary infection. As with ETT-aspirate sTREM-1, among those diagnosed with NVAP without extrapulmonary infection, serum sTREM-1 concentrations were higher, on average, in full-term than in preterm neonates.

Among neonates diagnosed with NVAP without extrapulmonary infection, the mean concentration of ETT-aspirate sTREM-1 was higher than that of serum sTREM-1; however, this was reversed among those without NVAP, but with extrapulmonary infection. This can be understood because of the difference in infection sites. In neonates not diagnosed with NVAP or extrapulmonary infection, the ETT-aspirate sTREM-1 concentration was higher, on average, than that of serum sTREM-1; however, the relatively low concentration of ETT-aspirate sTREM-1 in NVAP-negative cases indicate that the application of a mechanical ventilator may reduces the secretion of ETT-aspirate sTREM-1, probably due to sTREM-1 secreting cells (macrophages, neutrophils and others immune cells) in lung tissue were more than in serum (26). As we did not compare these concentrations to non-ventilated neonates, however, further research is required to identify such an influence.

Mean serum C-reactive protein and procalcitonin concentrations were also higher in infection cases; white blood cell count, however, was decreased in some infection cases. Similar pathogens were present in the ETT-aspirate of preterm and full-term neonates with NVAP (pneumoniae, *P. aeruginosa, A. baumannii* and *E. coli*); no pathogens were detected in ETT-aspirate of neonates without NVAP.

We identified ETT-aspirate sTREM-1 concentration as an indicator of NVAP, with an AUC of 0.986 (95% CI, 0.920–1.000) and a cutoff value of 228.0 pg/ml (sensitivity = 94.3%, specificity = 96.0%) in preterm neonates, and an AUC value of 0.938 (95% CI, 0.937–1.000) and a cutoff value of 245.5 pg/ml (sensitivity = 100.0%, specificity = 93.7%) in full-term neonates. These results are similar to those of a previous study (29), and demonstrated its potential utility for NVAP diagnosis. We also observed an increased mean serum sTREM-1 concentration in NVAP cases; however, its AUC, sensitivity, and specificity were lower than those of mean ETT-aspirate sTREM-1 concentration. One study revealed no statistically significant difference between ETT-aspirate and serum sTREM-1 concentrations in VAP patients (29), whereas others revealed a higher concentration of ETT-aspirate sTREM-1 than that in the serum (30, 31). Therefore, we also analyzed the ratio of ETT-aspirate to serum sTREM-1 concentration for its effectiveness as an indicator of NVAP. This ratio had lower AUC and specificity values (as low as 37.5% in full-term neonates), on average, than that of ETT-aspirate sTREM concentration. This suggests that the ETT-aspirate/serum sTREM-1 ratio is not an effective indicator of NVAP. Upon analysis of the other indicators, we discovered that serum C-reactive protein had an AUC of 0.947 (95% CI, 0.896–0.998) and a cutoff value of 2.88 ng/ml (sensitivity = 91.4%, specificity = 84.0%) in preterm neonates, and an AUC value of 0.915 (95% CI, 0.903–1.000) and a cutoff value of 6.25 ng/ml (sensitivity = 94.1%, specificity = 87.5%) in full-term neonates. Therefore, it may also be an effective indicator of NVAP. However, analysis of white blood cell count and serum procalcitonin concentration exhibited AUCs below 0.7 in each group. Upon multivariate logistic regression analysis, the optimal model for predicting NVAP was that incorporating both ETT-aspirate sTREM-1 and serum C-reactive protein concentration. This combination was a better predictor of NVAP than any single indicator.

All five indicators tested were present at lower levels on days 8 and 10 of ventilation in neonates who ultimately recovered, than in those who did not, and none of these markers improved notably from day 4 to 10 in patients who did not recover.

The major limitation of this study was the lack of baseline measurements for the indicators analyzed, i.e., measured before ventilator use. Furthermore, there is no data in terms of the standard range of ETT-aspirate and serum sTREM-1 concentrations in healthy preterm and full-term neonates. Finally, there are no gold standard criteria for the diagnosis of NVAP.

## Conclusion

We have demonstrated that the ETT-aspirate sTREM-1 and serum C-reactive protein concentrations may be useful for the diagnosis of NVAP in both preterm and full-term neonates. Further research is required to determine the generalizability of these results.

## Data Availability Statement

The original contributions presented in the study are included in the article/supplementary material, further inquiries can be directed to the corresponding author/s.

## Ethics Statement

The studies involving human participants were reviewed and approved by the First People's Hospital of Yongkang's institutional ethics committee (approval number: ykyy2018-03). Written informed consent to participate in this study was provided by the participants' legal guardian/next of kin. Written informed consent was obtained from the individual(s), and minor(s)' legal guardian/next of kin, for the publication of any potentially identifiable images or data included in this article.

## Author Contributions

JiaZ and LH conceived and designed the study. LH, JiaZ, YH, and YW performed the test. JiaZ, HL, and JinZ analyzed the data. JiaZ wrote the paper. All authors have read and approved the manuscript.

## Conflict of Interest

The authors declare that the research was conducted in the absence of any commercial or financial relationships that could be construed as a potential conflict of interest.

## Publisher's Note

All claims expressed in this article are solely those of the authors and do not necessarily represent those of their affiliated organizations, or those of the publisher, the editors and the reviewers. Any product that may be evaluated in this article, or claim that may be made by its manufacturer, is not guaranteed or endorsed by the publisher.
